# Retinoic acid receptor alpha drives cell cycle progression and is associated with increased sensitivity to retinoids in T-cell lymphoma

**DOI:** 10.18632/oncotarget.15441

**Published:** 2017-02-17

**Authors:** Xueju Wang, Surendra Dasari, Grzegorz S. Nowakowski, Konstantinos N. Lazaridis, Eric D. Wieben, Marshall E. Kadin, Andrew L. Feldman, Rebecca L. Boddicker

**Affiliations:** ^1^ Department of Laboratory Medicine and Pathology, Mayo Clinic, Rochester, Minnesota, United States of America; ^2^ Department of Pathology, China-Japan Union Hospital of Jilin Province, Changchun, Jilin Province, China; ^3^ Division of Biomedical Statistics and Informatics, Department of Health Sciences Research, Mayo Clinic, Rochester, Minnesota, United States of America; ^4^ Division of Hematology, Department of Medicine, Mayo Clinic, Rochester, Minnesota, United States of America; ^5^ Center for Individualized Medicine, Mayo Clinic, Rochester, Minnesota, United States of America; ^6^ Division of Gastroenterology and Hepatology, Department of Medicine, Mayo Clinic, Rochester, Minnesota, United States of America; ^7^ Department of Biochemistry and Molecular Biology, Mayo Clinic, Rochester, Minnesota, United States of America; ^8^ Department of Pathology and Laboratory Medicine, Rhode Island Hospital and Department of Dermatology, Roger Williams Medical Center, Providence, Rhode Island, United States of America

**Keywords:** T-cell lymphoma, retinoids, retinoic acid receptor alpha, all-trans retinoic acid, cell cycle, individualized medicine

## Abstract

Peripheral T-cell lymphomas (PTCLs) are aggressive non-Hodgkin lymphomas with generally poor outcomes following standard therapy. Few candidate therapeutic targets have been identified to date. Retinoic acid receptor alpha (RARA) is a transcription factor that modulates cell growth and differentiation in response to retinoids. While retinoids have been used to treat some cutaneous T-cell lymphomas (CTCLs), their mechanism of action and the role of RARA in CTCL and other mature T-cell lymphomas remain poorly understood. After identifying a PTCL with a RARA^R394Q^ mutation, we sought to characterize the role of RARA in T-cell lymphoma cells. Overexpressing wild-type RARA or RARA^R394Q^ significantly increased cell growth in RARA^low^ cell lines, while RARA knockdown induced G1 arrest and decreased expression of cyclin-dependent kinases CDK2/4/6 in RARA^high^ cells. The retinoids, AM80 (tamibarotene) and all-*trans* retinoic acid, caused dose-dependent growth inhibition, G_1_ arrest, and CDK2/4/6 down-regulation. Genes down-regulated in transcriptome data were enriched for cell cycle and G1-S transition. Finally, RARA overexpression augmented chemosensitivity to retinoids. In conclusion, RARA drives cyclin-dependent kinase expression, G_1_-S transition, and cell growth in T-cell lymphoma. Synthetic retinoids inhibit these functions in a dose-dependent fashion and are most effective in cells with high RARA expression, indicating RARA may represent a therapeutic target in some PTCLs.

## INTRODUCTION

Peripheral T-cell lymphomas (PTCLs) are aggressive non-Hodgkin lymphomas of mature T-cell origin that demonstrate marked clinical, pathological, and molecular heterogeneity, with over 20 subtypes currently recognized by the World Health Organization [1]. Outcomes generally are poor following standard combination chemotherapy regimens, most commonly cyclophosphamide, doxorubicin (hydroxydaunorubicin), Oncovin (vincristine), and prednisone (CHOP) [2]. Although these data indicate a pressing need for new therapeutic approaches in PTCL, attempts to improve outcomes using alternative chemotherapy regimens have been disappointing. Targeted therapies offer promise, but data on drug and patient selection are limited [3]. Therefore, the identification and validation of candidate therapeutic targets in PTCL is critical to improving outcomes in this disease.

Retinoic acid receptor alpha (RARA) is a transcription factor that forms heterodimers with retinoid X receptor (RXR) [4]. These heterodimers bind to DNA motifs known as retinoic acid response elements (RAREs) and regulate gene transcription upon interaction with the natural ligand, retinoic acid, resulting in the regulation of genes involved in cellular growth and differentiation. Although it was originally thought that ligand-binding to RARA resulted in transcriptional activation, chromatin immunoprecipitation-sequencing and transcriptome profiling have revealed roles for RARA as both a repressor and activator of transcription [5, 6]. Retinoic acid has demonstrated anti-proliferative effects in many tumor models, and as such, retinoic acid receptors (RARs) have been targeted therapeutically through the use of natural and synthetic retinoids.

Retinoid therapy has been used most notably for the treatment of acute promyelocytic leukemia, a myeloid neoplasm expressing RARA fusion proteins [4, 7]. In addition, retinoids have been used effectively in some cutaneous T-cell lymphomas (CTCLs), a group of mature T-cell lymphomas originating in the skin, for which the synthetic RXR retinoid, bexarotene, is approved by the United States Food and Drug Administration (FDA) as a second-line therapy [8]. Response rates to RAR and RXR retinoids as monotherapy in CTCL are around 50% [9, 10]. Though experience remains limited, occasional partial or complete responses to retinoids also have been observed in relapsed/refractory systemic PTCLs [11, 12]. However, the mechanism(s) of action of retinoids in PTCL, the specific role of RARA, and a means to identify patients most likely to respond to retinoids are unknown.

Individualized medicine approaches have been employed to use the results of high-throughput sequencing to identify drug-target combinations specific for each patient. Recently, we evaluated a patient with PTCL in the Mayo Clinic Center for Individualized Medicine, whose tumor bore a non-synonymous somatic mutation, RARA^R394Q^, in the ligand-binding region of the *RARA* gene (non-synonymous mutations summarized in Supplementary Table 1). Since this mutation had not been previously reported and the role of RARA in PTCL had not been characterized, we investigated the role of RARA in the growth and chemosensitivity to retinoids in T-cell lymphoma cells.

## RESULTS

### Wild-type and mutant RARA proteins drive T-cell lymphoma cell growth

**Figure 1:**
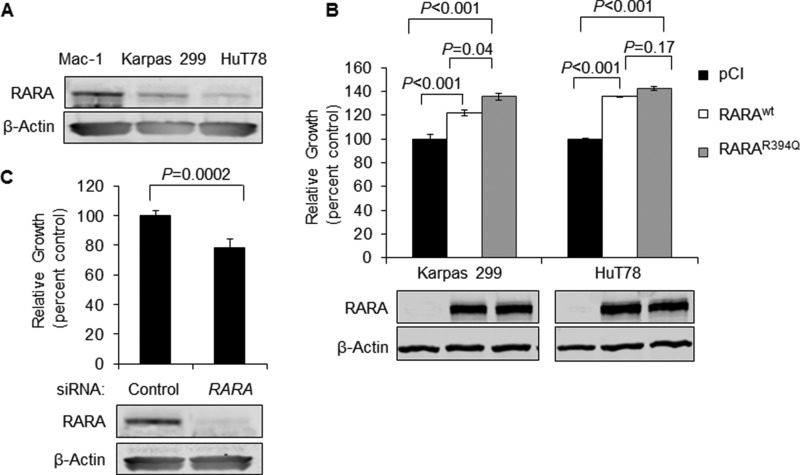
Overexpression of RARA^wt^ or RARA^R394Q^ drives T-cell lymphoma cell growth (**A**) Native RARA is expressed strongly in Mac-1 and to a lesser degree in Karpas 299 and HuT78 cell lines. (**B**) Cell growth is increased upon overexpression of RARA^wt^ or RARA^R394Q^ in Karpas 299 and HuT78 cell lines with low native RARA expression. (**C**) Knockdown of RARA inhibits cell growth in Mac-1 cells with high native RARA expression. RARA, retinoic acid receptor alpha; wt, wild-type; siRNA, small interfering RNA.

To investigate the role of wild-type RARA (RARA^wt^) and RARA^R394Q^, we utilized three mature T-cell lymphoma cell lines (see Materials and Methods) with varied native RARA expression: one RARA^high^ cell line (Mac-1) and two RARA^low^ cell lines (Karpas 299 and HuT78; Figure 1A). We used the two RARA^low^ cell lines to examine the effects of overexpressing RARA^wt^ or RARA^R394Q^ on cell growth, compared to an empty-vector control (pCI). RARA^wt^ increased growth of Karpas 299 by 22% (*P* < 0.001) and of HuT78 by 36% (*P* < 0.001), while RARA^R394Q^ increased growth of Karpas 299 by 36% (*P* < 0.001) and of HuT78 by 42% (*P* < 0.001; Figure 1B). The difference in the increase in growth between RARA^R394Q^ and RARA^wt^ was statistically significant in Karpas 299 (*P* = 0.04) but not in HuT78 (*P* = 0.17). Because both RARA^R394Q^ and RARA^wt^ increased cell growth but the R394Q mutation conferred only a mild growth advantage over wild-type, we focused our efforts preferentially on understanding the growth-promoting role of RARA in general, rather than characterizing the specific effects of the R394Q mutation on RARA function. In keeping with the growth-promoting role of RARA, siRNA knockdown of *RARA* in RARA^high^ Mac-1 cells resulted in a 22% inhibition of cell growth (*P* = 0.0002; Figure 1C).

### RARA drives cyclin-dependent kinase expression and G_1_-S transition in T-cell lymphoma cells

**Figure 2:**
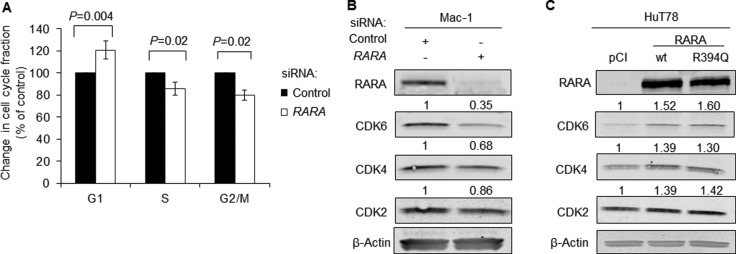
*RARA* drives expression of cyclin-dependent kinases (**A**) Knockdown of *RARA* causes G_1_ cell cycle arrest (*P* = 0.004) in Mac-1 cells. (**B**) Expression of the regulators of cell cycle progression, CDK6, CDK4, and to a lesser extent, CDK2, is inhibited by *RARA* knockdown in Mac-1 cells. (**C**) Expression of CDK6, CDK4, and CDK2 is increased following overexpression of RARA^wt^ and RARA^R394Q^ in HuT78 cells. RARA, retinoic acid receptor alpha; wt, wild-type; siRNA, small interfering RNA; CDK, cyclin-dependent kinase.

Having identified a role for RARA in driving T-cell lymphoma cell growth, we next examined the effect of RARA on the cell cycle. siRNA knockdown of *RARA* in RARA^high^ Mac-1 cells resulted accumulation of cells in G_1_ (120% of control, *P* = 0.004), with corresponding decreases in the fractions of cells in S-phase and G_2_/M (*P* = 0.02; Figure 2A). To explore this finding further, we evaluated the expression of the cyclin-dependent kinases (CDKs), CDK6, CDK4, and CDK2, which are involved in the regulation of the G_1_-S transition [13]. Indeed, *RARA* knockdown in Mac-1 cells inhibited CDK6, CDK4, and CDK2 protein expression by 65%, 32%, and 14%, respectively (Figure 2B). Correspondingly, overexpression of RARA^wt^ increased CDK6, CDK4, and CDK2 protein expression by 52%, 39%, and 39% respectively; overexpression of RARA^R394Q^ caused similar increases in CDK expression (60%, 30%, and 42% respectively; Figure 2C).

### Retinoids cause RARA degradation and cell-cycle arrest in T-cell lymphoma cells

Because we showed that RARA drove T-cell lymphoma cell growth and cell-cycle progression, we next examined the ability of retinoids to reverse these effects. We evaluated the activity of two retinoids that act as ligands for RARA. All-*trans* retinoic acid (ATRA) is a ligand for all RARs [14], while the synthetic retinoid, AM80 (4-[(5,6,7,8-tetrahydro-5,5,8,8-tetramethyl-2-naphthyl)carbamoyl]benzoic acid or tamibarotene), preferentially targets RARA and retinoic acid receptor beta (RARB). However, *RARB* is not expressed in the cell lines used in this study (Supplementary Figure 1). Therefore, we used AM80 as a relatively specific RARA ligand and ATRA as a less specific ligand also predicted to target retinoic acid receptor gamma (RARG), which is expressed in our cell lines, albeit at somewhat lower levels than RARA. In addition, we evaluated the ability of the RXR ligand, bexarotene, to target RARA because of its clinical application in cutaneous T-cell lymphoma.

**Figure 3:**
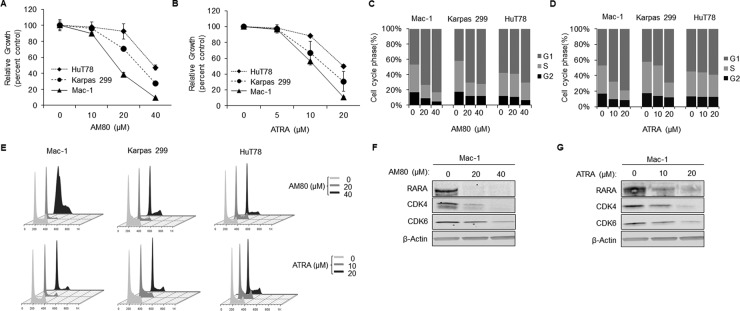
Retinoids cause G_1_-arrest and concurrent inhibition of RARA and CDK4/6 expression (**A**, **B**) AM80 and ATRA cause dose-dependent growth inhibition in T-cell lymphoma cells that is proportional to native RARA expression (as shown in Figure 1A). (**C**, **D**) AM80 and ATRA cause G_1_ arrest in T-cell lymphoma cells that is proportional to native RARA expression. (**E**) Representative cell cycle plots of T-cell lymphoma cells after treatment with AM80 or ATRA. (**F**, **G**) AM80 and ATRA cause RARA degradation and down-regulation of CDK4 and CDK6 in RARA^high^ Mac-1 cells. RARA, retinoic acid receptor alpha; CDK, cyclin-dependent kinase; ATRA, all-*trans* retinoic acid.

Treatment of T-cell lymphoma cell lines with AM80, ATRA, or bexarotene resulted in dose-dependent inhibition of cell growth (Figures 3A, 3B and Supplementary Figure 3A). Importantly, the RARA^high^ cell line, Mac-1, was most chemosensitive to retinoids; the RARA^low^ cell lines, Karpas 299 and HuT78, were less chemosensitive, with the cell line expressing the lowest level of RARA protein, HuT78, demonstrating the least chemosensitivity. Cell-cycle analysis revealed dose-dependent G_1_ arrest following treatment with AM80 or ATRA (Figure 3C–3E). Again, these effects were most pronounced in the RARA^high^ cell line, Mac-1. RARA ligands previously have been shown to lead to RARA degradation through the ubiquitin-proteasome pathway [15]. Consistent with this known process, RARA protein was markedly reduced after treatment with AM80, ATRA, or bexarotene (Figure 3F, 3G and Supplementary Figure 3B). Furthermore, treatment with either AM80 or ATRA resulted in dose-dependent decreases in the expression of CDK4 and CDK6 proteins.

### Retinoids suppress a cell cycle gene expression program in T-cell lymphoma cells

**Figure 4:**
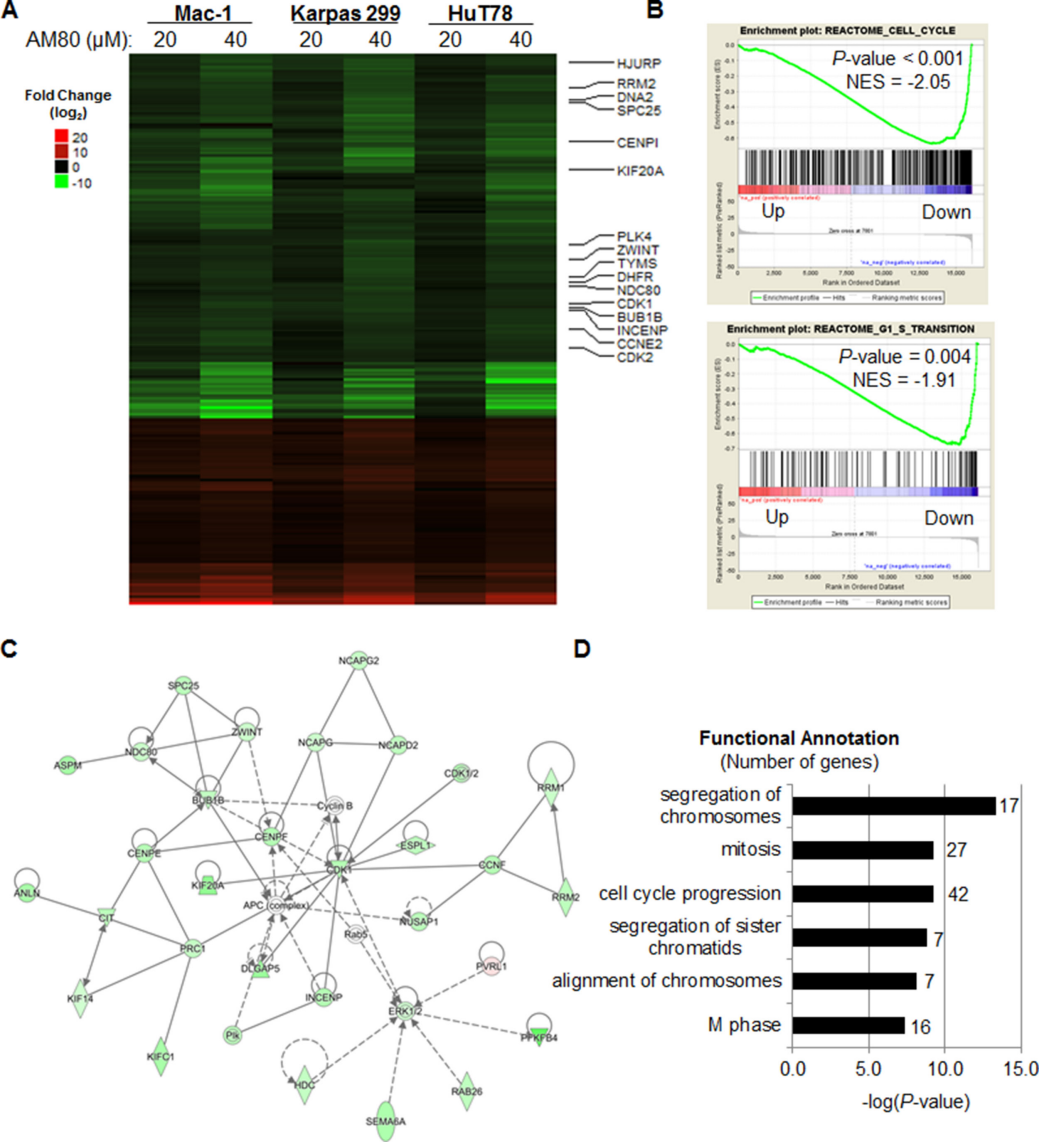
The RARA-specific retinoid, AM80, down-regulates expression of cell cycle gene in T-cell lymphoma cells (**A**) AM80 treatment results in a consistent gene expression signature across T-cell lymphoma cell lines that includes down-regulation of multiple cell cycle genes. The heat map displays log_2_ fold changes from control (0 μM dose) at 24 hours. (**B**) Gene set enrichment analysis (GSEA) plots show enrichment for cell cycle and G_1_-S transition gene sets in genes down-regulated by AM80. (**C**) Pathway analysis of genes differentially expressed upon AM80 treatment identifies a top regulatory network (score = 56) comprising cell cycle, cellular assembly and organization, and DNA replication-associated genes. (**D**) Functional annotation of genes differentially expressed by AM80 identifies cell cycle-related functions as top hits. NES, Normalized Enrichment Score.

To characterize the effects of retinoids on T-cell lymphoma cells further, we performed RNA sequencing and analyzed the transcriptomes of Mac-1, Karpas 299, and HuT78 cell lines treated with AM80, ATRA, or bexarotene at varying doses. We then identified genes consistently up- or down-regulated in a dose-dependent manner compared to vehicle-treated control cells. Of 235 genes differentially expressed after AM80 treatment, 156 (66%) were down-regulated and 79 (34%) were up-regulated (Figure 4A). Down-regulated genes included several key cell cycle regulators including *CDK1*, *CDK2*, *TYMS* (thymidylate synthetase), and *CCNE2* (cyclin E2). Gene set enrichment analysis (GSEA) confirmed that genes down-regulated by AM80 were highly enriched for regulators of the cell cycle (*P* < 0.001) and, more specifically, the G_1_-S transition (*P* = 0.004; Figure 4B). In addition, comprehensive network analysis identified that the most prominent network (score = 56) in the list of genes differentially expressed by AM80 was composed of genes associated with cell cycle, cellular assembly and organization, and DNA replication (Figure 4C). Finally, functional annotation of differentially expressed genes identified cellular processes involving cell replication and cell cycle progression (Figure 4D). Taken together with the relative specificity of AM80 for RARA, these findings strongly support and extend our *in vitro* functional observations that RARA drives cell cycle progression and, more specifically, G_1_-S transition, in T-cell lymphoma cells.

The pan-RAR agonist, ATRA, as well as the RXR agonist, bexarotene, also down-regulated expression of genes involved in cell cycle regulation, including genes identical to those down-regulated by AM80 (Supplementary Figures 2A and 3C). However, consistent with the broader array of RAR and RXR targets for ATRA and bexarotene, respectively, the functions of the differentially expressed genes were more diverse. Network analysis for both ATRA and bexarotene identified the top networks to involve lipid metabolism and small molecule biochemistry rather than cell cycle (Supplementary Figure 2B and 2D).

### RARA overexpression increases chemosensitivity to the synthetic retinoid, AM80

**Figure 5:**
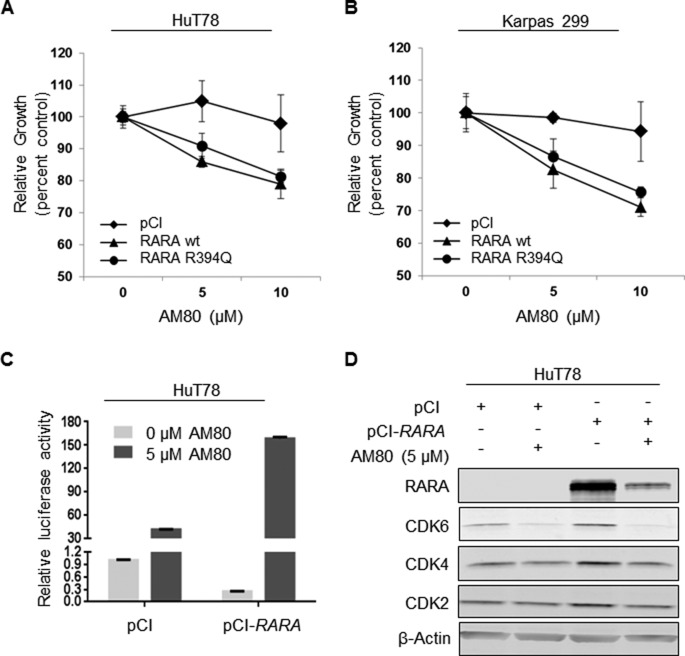
RARA overexpression increases chemosensitivity to AM80 (**A**, **B**) Overexpression of either RARA^wt^ or RARA^R394Q^ increases chemosensitivity of (A) HuT78 and (B) Karpas 299 cells to AM80. The presence of the R394Q mutation does not reduce chemosensitivity significantly. (**C**) RARA^wt^ overexpression increases retinoic acid response element (RARE) activity (indicated by luciferase) in HuT78 cells treated with AM80. (**D**) RARA^wt^ overexpression increases the degree of CDK2, CDK4 and CDK6 inhibition caused by AM80. wt, wild-type; CDK, cyclin-dependent kinase.

Because of our observation that the cell line with the highest native RARA expression, Mac-1, exhibited the most chemosensitivity to retinoids, we explored the role of RARA overexpression on retinoid chemosensitivity in cell lines with lower RARA expression. Using doses of AM80 to which the RARA^low^ cell lines, HuT78 and Karpas 299, were relatively resistant, we found that overexpression of RARA^wt^ significantly increased chemosensitivity compared to empty vector control (HuT78: 5 μM, 18% growth inhibition, *P* = 0.01; 10 μM, 19% growth inhibition, *P* = 0.04; and Karpas 299: 5 μM, 16% growth inhibition, *P* = 0.04; 10 μM, 23% growth inhibition, *P* = 0.02; Figure 5A, 5B). Overexpressing RARA^R394Q^ conferred slightly less chemosensitivity than RARA^wt^ in both cell lines, but these differences were not statistically significant.

Because RARs regulate transcription by binding to RARE motifs in target gene promoters, we examined the effect of RARA^wt^ overexpression with and without AM80 treatment on RARE activity using a luciferase assay. As expected, in the absence of AM80, RARE activity was lower in HuT78 cells overexpressing RARA than in cells with low basal RARA expression (Figure 5C). However, AM80 increased RARE activity in RARA-overexpressing cells to a much greater degree (382% of control) than in RARA^low^ cells. Finally, RARA^wt^ overexpression augmented the degree of CDK2, CDK4, and CDK6 inhibition caused by AM80 treatment (Figure 5D). Taken together, these findings suggest that T-cell lymphoma cells expressing high levels of RARA are particularly chemosensitive to RARA-directed retinoids, and that measuring RARA protein expression—but not necessarily identifying *RARA* mutations—might be used predict this degree of chemosensitivity.

## DISCUSSION AND CONCLUSIONS

In this study, we used a combined approach of transcriptome sequencing, functional studies, and inhibitor assays to characterize the role of RARA in mature T-cell lymphoma cells. Following identification of a RARA^R394Q^ mutation in a PTCL patient being evaluated in an individualized medicine clinic, we demonstrated that both RARA^R394Q^ and RARA^wt^ accelerated growth of T-cell lymphoma cells, with modestly enhanced function of the mutant compared to the wild-type form. We then demonstrated that the growth induction caused by RARA was due to promotion of the cell cycle, specifically G_1_-S transition. Finally, we demonstrated that these effects could be reversed by retinoids that target RARA, and that indeed high RARA expression increased chemosensitivity to these agents. These findings suggest the possibility of therapeutic approaches including retinoids for some patients with PTCL, and the potential use of determining RARA expression to identify those patients most likely to derive benefit from retinoid therapy.

Clinical genomic sequencing approaches to individualize treatment have become an important new strategy for the management of patients with cancer and other diseases [16]. While sequence data from some patients point to genetic abnormalities that encode protein products known to be targetable with existing drugs, the significance of many findings remains unclear, prompting functional investigations in disease-specific experimental models [3]. Here, we examined the potential role of RARA^R394Q^ in T-cell lymphoma. This mutation was chosen as a candidate for further study because amino acid R394 of RARA has been demonstrated to be important in retinoid binding [17]. However, we observed only modest functional differences between RARA^wt^ or RARA^R394Q^ overexpression in T-cell lymphoma cells either in the presence or absence of retinoids. Interestingly, another substitution affecting this same amino acid, RARA^R394W^, was reported in two patients with relapsed acute promyelocytic leukemia with PML-RARA fusions, and was shown to be associated with only modest effects on ATRA binding and RARE reporter transactivation; however, a 12-nucleotide deletion in this same region, ΔR394-L398/394M, had pronounced effects in these assays [18]. Therefore, amino acid substitutions alone at RARA R394 do not appear to have immediate clinical relevance with regard to retinoid chemosensitivity.

Although the role of RARA^R394Q^ remains unclear, overexpression of either RARA^R394Q^ or RARA^wt^ increased T-cell lymphoma cell growth significantly, and enhanced chemosensitivity was observed in RARA^high^ cells as well as RARA^low^ cells experimentally manipulated to overexpress RARA. Importantly, clinical responses to retinoids have been reported in various T-cell malignancies, including PTCL, CTCL, and adult T-cell leukemia (ATL) [9–12, 19–22]. For example, Cheng et al reported 5 complete responses and one partial response among 12 PTCL patients treated with 13-cis retinoic acids [12]. We found RARA to be the predominantly expressed RAR in PTCL cell lines, but also noted that its expression varied among the lines tested. As predicted, the relationship between RARA expression and chemosensitivity was more marked for the relatively RARA-specific retinoid, AM80, than for the pan-RAR ligand ATRA or the RXR-ligand bexarotene. Of note, a relationship between RARA expression and AM80 sensitivity also has been reported in acute myeloid leukemia cells [23]. Taken together, these data suggest the possibility that, while clinical response rates to retinoids in patients with systemic PTCLs have been modest, these response rates might be improved by using RARA expression as a biomarker to select patients more likely to be chemosensitive.

Our data demonstrate a critical role for RARA in cell cycle regulation of T-cell lymphoma cells, and particularly in G_1_-S transition. In myeloid cells, retinoic acid has been shown to induce G_1_ arrest with corresponding downregulation of cyclins D and E and decreased CDK activity [24–26]. Similarly, AM80 has been shown to cause G_1_ arrest in ATL cells [27]. Although chromatin immunoprecipitation studies have demonstrated binding of RARA to *CDK2* and *CDK6*, a direct effect on transcriptional regulation has not been demonstrated; thus, inhibition of CDKs may occur as an indirect, downstream effect of retinoid activity [28, 29]. Of note, CDKs including CDK6 play a critical role in physiological development and tumorigenesis of thymocytes [30]. In addition, recurrent amplifications of *CDK6* as well as deletions of *CNDK2A* encoding the CDK4/6 inhibitor, p16^INK4A^, have been reported in PTCLs [31–33]. Clinical CDK4/6 inhibitors have shown activity in B-cell lymphomas and other cancers [13], and might be an alternative means to block CDK up-regulation in RARA^high^ T-cell lymphomas.

## MATERIALS AND METHODS

### Retinoids

ATRA (Sigma-Aldrich, Milwaukee, WI, USA), AM80 (Tocris, Ellisville, MS, USA), and bexarotene (Selleck Chemicals, Houston, TX, USA) were dissolved in dimethyl sulfoxide and stock solutions and stored at −80°C. Retinoids were protected from light during handling.

### Cell lines, culture conditions, and transfection

Karpas 299 (ALK-positive anaplastic large cell lymphoma, a PTCL subtype) and HuT78 (CTCL) cell lines were obtained from ATCC, Gaithersburg, MD, USA. Mac-1 (developed by M.E.K.) was derived from circulating CTCL cells from a patient with multiple T-cell neoplasms [34]. Karpas 299 and Mac-1 were maintained in RPMI 1640 (Gibco, Grand Island, NY, USA) containing 10% fetal bovine serum (FBS; Clontech Laboratories, Inc, Mountain View, CA, USA) and 1% penicillin/streptomycin (Gibco, Grand Island, NY, USA). HuT78 was maintained in Iscove's Modified Dulbecco's Medium (Gibco, Grand Island, NY, USA) containing 15% FBS and 1% penicillin/streptomycin. All cell lines were electroporated with 300V for 10 msec in antibiotic-free medium using the ECM 830 electroporation system (BTX Harvard Apparatus).

### siRNA gene knockdown

Control (AllStars Negative Control; Qiagen, Valencia, CA, USA) and *RARA* (ON-TARGETplus SMARTpool; GE Dharmacon, Waltham, MA, USA) siRNAs were transfected by electroporation and protein expression was assessed at 72 hours. Pooled *RARA* siRNA sequences were: GCAAAUACACUACGAACAA; CCA AGGAGUCUGUGAGAAA; GAGCAGCAGUUCUGA AGAG; GAACAACGUGUCUCUCUGG.

### Expression plasmids

Human *RARA* was subcloned from pcDNA6-*RARA* (Plasmid# 35555 [35], Addgene, Cambridge, MA, USA; NheI and XbaI restriction sites) into pCI (Promega, Madison, WI, USA). The R394Q mutation was introduced into pCI-*RARA* using site-directed mutagenesis (QuickChange II, Agilent Technologies, Santa Clara, CA, USA). Expression vectors were transiently transfected into Karpas 299 and HuT78 cells by electroporation and protein expression was assessed at 48hours. For retinoid treatment, cells were cultured in media containing 2% charcoal-filtered FBS immediately following transfection, and cell treatments were administered after a 4-hour post-electroporation recovery period.

### Cell growth

The CellTiter Aqueous Non-Radioactive Cell Proliferation Assay Kit (MTS; Promega) was used to assess cell growth following the manufacturer's instructions. Briefly, 10,000 cells per well were seeded in a 96-well plate and cultured in RPMI containing 2% charcoal-stripped FBS and indicated retinoid concentrations (Gibco, Grand Island, NY, USA) for 72 hours. At the end of the treatment period, the MTS reagent was added, cells were incubated an additional 2–4 hours, and absorbance was measured at 490 nanometers.

### Cell cycle analysis

Cell cycle analysis was performed 72 h post- transfection or drug treatment. Approximately 1 × 10^6^ cells were pelleted, washed in phosphate-buffered saline, and fixed in 75% ethanol overnight at 4°C. Cells then were washed in phosphate-buffered saline, re-suspended in a 1× saponin-based permeabilization buffer and wash reagent (Thermo Fisher Scientific, Waltham, MA, USA), and stained with FxCycle™ Far Red stain (Thermo Fisher) for 30 minutes at room temperature. Samples were evaluated on a FACSCalibur cytometer (Becton Dickinson, Mountain View, CA, USA). Data were analyzed using FlowJo 7.6.5 software (Tree Star Inc., Ashland, OR, USA).

### Immunoblotting

Immunoblotting was performed as previously described [36] using antibodies to RARA (sc-551, Santa Cruz Biotechnology, Inc, Santa Cruz, CA, USA), CDK2 (78B2/ #2546, Cell Signaling Technology, Danvers, MA, USA), CDK4 (D9G3E/#12790, Cell Signaling), CDK6 (D4S8S/#13331, Cell Signaling) or β-actin (NB600-501, Novus Biologicals, LLC, Littleton, CO, USA). Proteins were detected using goat anti-mouse (sc-2005, Santa Cruz) or donkey anti-rabbit (NA934, Amersham, Little Chalfont, United Kingdom) antibodies and West Pico Super Signal chemiluminescence (Thermo Fisher), or using IRDye 800CW and 680RD antibodies (LI-COR Biosciences, Lincoln, NE, USA) on the LI-COR Odyssey CLx Imaging System.

### Luciferase reporter assays

RARE reporter (Cignal RARE reporter; SABiosciences, Frederick, MD, USA) and pCI empty vector or pCI-*RARA* (wild-type) were co-transfected into HuT78 cells by electroporation. After recovery for 24 hours, the transfected cells were treated with AM80 or vehicle (dimethyl sulfoxide) in RPMI containing 2% charcoal-stripped FBS for 24 hours. Luciferase expression was measured using the Dual-Luciferase Reporter Assay System (Promega) following manufacturer's protocol using the Centro XS3 LB 960 microplate luminometer (Berthold Technologies, Zug, Switzerland) as previously described [36]. Total protein concentrations of each lysate were used for normalization.

### Transcriptome analysis

Mac-1, Karpas 299 and HuT78 cells were treated with AM80 (0 μM, 20 μM, or 40 μM), ATRA (0 μM, 10 μM, or 20 μM), or bexarotene (0 μM, 10 μM, or 20 μM) in medium containing 2% charcoal-stripped FBS. Cell pellets were collected at 24 hours and immediately stored at −80°C pending RNA extraction. RNA sequencing was performed as previously described [3]. Briefly, following extraction of total RNA, mRNA libraries were constructed (Illumina, TruSeq Technology, San Diego, CA, USA) and 100 base-pair paired-end sequencing was performed on an Illumina HiSeq instrument. The Sequencing data were mapped to hg19 and genes with a minimum of 100 reads in at least one sample were retained for analysis. Dose-dependent effects of each drug were determined using paired *t*-tests (EdgeR) for medium-versus-low and high-versus-medium comparisons. Genes were considered differentially expressed if they met fold-change and *P*-value criteria (log_2_ fold change ≥ 0.5 or ≤ −0.5 and *P*-value ≤ 0.05) for both comparisons. GSEA was performed on a ranked dataset generated by the product of the fold-change sign and –log(*P*-value) for the 0 μM versus 40 μM AM80 comparison [37]. Pathway analysis was performed using Ingenuity Pathway Analysis software (Qiagen, Redwood City, CA, USA) on differentially expressed genes.

### Statistical analysis

Statistical analysis was performed using Student's *t*-test. Statistical significance was defined as *P* < 0.05.

## SUPPLEMENTARY MATERIALS FIGURES AND TABLE


