# Supplementary material for: Retinoic acid receptor alpha drives cell cycle progression and is associated with increased sensitivity to retinoids in T-cell lymphoma
Source: Oncotarget. 2017 Feb 17;8(16):26245–55. doi: 10.18632/oncotarget.15441 (PMC5432253; doi:10.18632/oncotarget.15441)
Supplement: Supplementary file 1 [file oncotarget-08-26245-s001.pdf]

Retinoic acid receptor alpha drives cell cycle progression and is associated with increased sensitivity to retinoids in T-cell lymphoma

Supplementary Materials

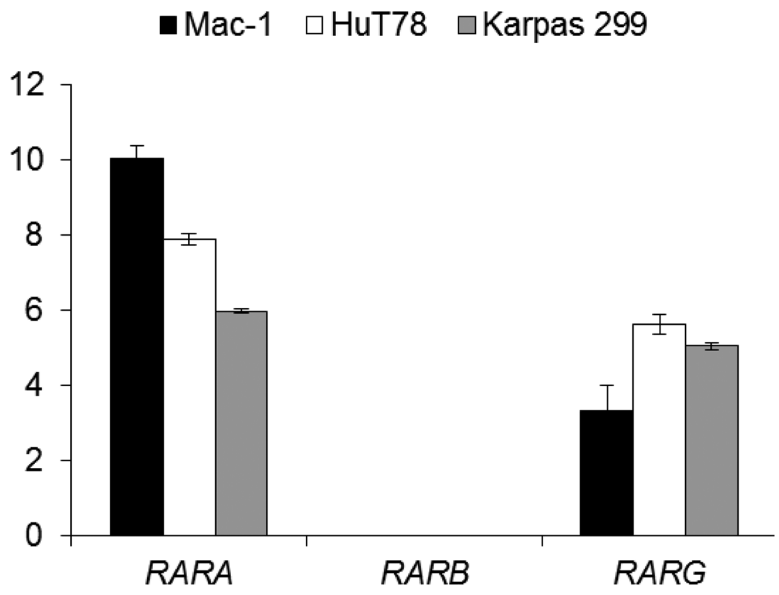

Supplementary Figure 1: Average expression of retinoic acid receptor genes in T-cell lymphoma cell lines. Expression of *RARA* is highest in Mac-1, similar to protein levels shown in Figure 1A. *RARB* is not expressed. *RARG* is expressed at somewhat lower levels than *RARA*. Data are from transcriptome analysis. RPKM, reads per kilobase per million mapped reads.

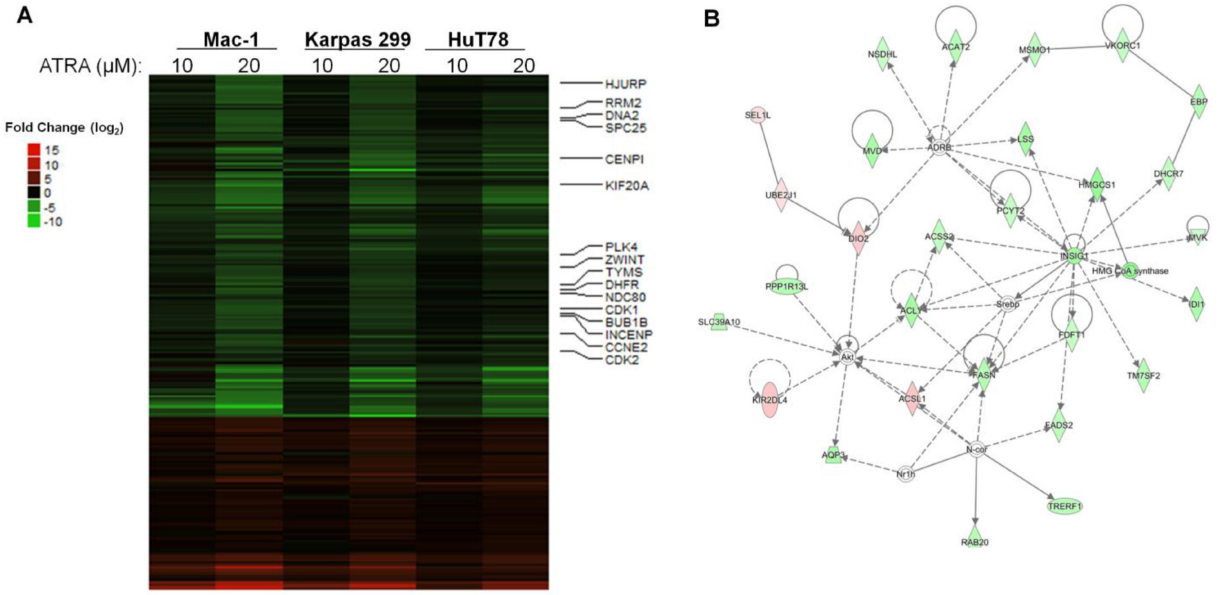

Supplementary Figure 2: Effect of ATRA on gene expression in T-cell lymphoma cells. (**A**) All-*trans* retinoic acid (ATRA) treatment results in a gene expression signature across T-cell lymphoma cell lines that includes down-regulation of cell cycle genes also seen after AM80 treatment (as shown in Figure 4). The heat map displays $\log_2$ fold changes from control (0 µM dose). The gene list is derived from the 235 genes differentially expressed upon AM80 treatment (as shown in Figure 4A). (**B**) Pathway analysis of genes differentially expressed upon ATRA treatment identifies a top regulatory network comprising lipid metabolism and small molecule biochemistry-associated genes.

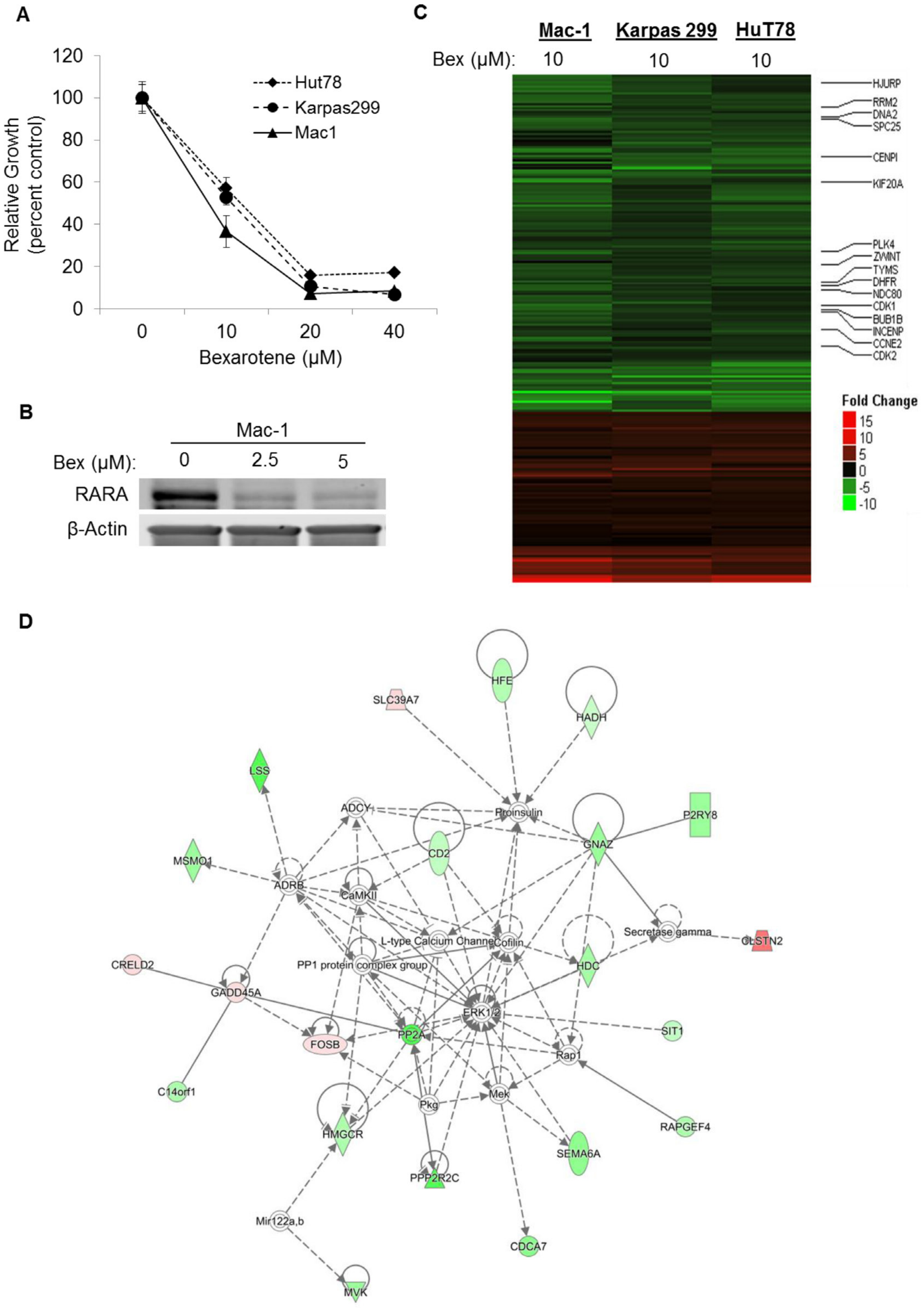

Supplementary Figure 3: Effect of bexarotene on gene expression in T-cell lymphoma cells. (**A**) Growth of Mac-1, Karpas 299, and HuT78 T-cell lymphoma cell lines is inhibited by bexarotene in a manner proportional to baseline RARA expression (see Figure 1A). (**B**) RARA protein decreases in response to bexarotene treatment in Mac-1 cells. (**C**) Bexarotene (Bex) treatment results in a gene expression signature across T-cell lymphoma cell lines that is similar to that seen after AM80 treatment (as shown in Figure 4), including down-regulation of cell cycle-related genes. The heat map displays $\log_2$ fold changes from control (0 µM dose). The gene list is derived from the 235 genes differentially expressed upon AM80 treatment (as shown in Figure 4A). (**D**) Pathway analysis of genes differentially regulated by bexarotene at 24 h identified a top regulatory network characterized by genes involved in lipid metabolism.

Supplementary Table 1: Non-synonymous somatic mutations identified in peripheral T-cell lymphoma patient sequenced in the Mayo Clinic Center for Individualized Medicine

Gene Name	Mutation
CDK12	L1457P
GTSE1	P191fs
HGF	G323R
IRF4	A163V
RARA	R394Q
MAPK1	R77H
NF1	W221*
PCLO	E2370D, M2542I, H3122Y, G4263V
PCD11	R1750Q
PRKDC	T3198I
RHOA	C16W
LRP1B	D1162N
SETD2	R472H
TBL1XR1	P89T
TP53	V157G
WT1	A61E
ZMYM3	P583S
